# Unlocking the Power of Magnesium: A Systematic Review and Meta-Analysis Regarding Its Role in Oxidative Stress and Inflammation

**DOI:** 10.3390/antiox14060740

**Published:** 2025-06-16

**Authors:** Violeta Cepeda, Marina Ródenas-Munar, Silvia García, Cristina Bouzas, Josep A. Tur

**Affiliations:** 1 Research Group on Community Nutrition and Oxidative Stress, University of the Balearic Islands-IUNICS, Guillem Colom Bldg, Campus, E-07122 Palma de Mallorca, Spaincristina.bouzas@uib.es (C.B.); 2 Health Research Institute of the Balearic Islands (IdISBa), 07120 Palma de Mallorca, Spain; 3 CIBER Fisiopatología de la Obesidad y Nutrición (CIBEROBN), Instituto de Salud Carlos III (ISCIII), 28029 Madrid, Spain

**Keywords:** Mg supplementation, antioxidant, inflammation, oxidative stress, meta-analysis

## Abstract

Magnesium plays a crucial role in over 300 enzymatic reactions related to energy production, muscle contraction, and nerve function. Given its essential biological functions and increasing prevalence of suboptimal intake, magnesium supplementation has gained attention for its potential health benefits, particularly in mitigating oxidative stress and inflammation. This systematic review and meta-analysis aimed to evaluate the antioxidant effects of dietary and supplemental magnesium on several biomarkers related to oxidative stress and inflammation. A systematic search of studies published from 2000 to 2025 identified 28 relevant articles, including both animal and human studies. The meta-analysis assessed the effects of magnesium supplementation on oxidative stress biomarkers such as nitric oxide (NO), total antioxidant capacity (TAC), malondialdehyde (MDA), glutathione (GSH), and C-reactive protein (CRP). While results showed a statistically significant reduction in CRP levels, suggesting an anti-inflammatory effect, no conclusive impact on oxidative stress biomarkers was observed. The findings highlight magnesium’s potential role in inflammation regulation, though its direct antioxidant effects remain uncertain. Further high-quality clinical trials are needed to clarify the impact of magnesium supplementation on oxidative stress and to explore its broader health implications.

## 1. Introduction

Magnesium (Mg) is an essential mineral involved in over 600 enzymatic reactions, including energy production, muscle contraction, and nerve transmission. Despite being present in many common foods, suboptimal magnesium intake is increasingly frequent due to modern dietary habits, soil depletion, and certain medical conditions that impair absorption or increase excretion [1,2,3], and around 200 enzymes more in which magnesium may function as an activator [4,5]. Consequently, Mg supplementation has gained popularity as a strategy to support overall health and address deficiencies [1,6].

In recent years, magnesium supplementation has gained popularity as a perceived solution to various physiological disorders. However, the growing popularity of magnesium supplements, in most cases, has not been accompanied by consistent clinical evidence. This has led to a widespread public perception of its benefits, often in the absence of a thorough evaluation of individual needs, clinical follow-up when supplementation has started, or solid scientific evidence to support such claims.

Accurately assessing magnesium status remains a clinical challenge. Since only about 1% of the body’s magnesium is found in the bloodstream, standard serum tests may not reliably reflect total body stores. The majority of Mg is stored in bones, muscles, and soft tissues, limiting the diagnostic value of routine blood tests [7].

For this reason, the practice of recommending magnesium supplementation without proper clinical evaluation may be cause for concern. While magnesium is essential for numerous physiological functions, indiscriminate supplementation may not be harmless for everyone. Moreover, excessive magnesium intake may interact with certain medications, such as antibiotics or diuretics [8], potentially altering their efficacy and, although less likely, may aggravate existing chronic diseases, such as kidney failure, or cause serious adverse effects such as hypotension, bradycardia, severe diarrhoea, or neuromuscular depression, among others [7]. It is important to highlight that there is currently no clear consensus regarding the optimal dosage and posology for magnesium supplementation, particularly in relation to the specific salt or formulation used.

In parallel, while many studies focus on the role of magnesium in cardiovascular or neuromuscular health, there is less clarity regarding its potential role in oxidative stress modulation, which is a mechanism linked to the development of chronic diseases.

Yet, the available data are scattered, often focused on disease-specific interventions or using magnesium in combination with other compounds, making it difficult to isolate its specific effects.

Given the increasing prevalence of Mg supplementation and the growing interest in its potential effects, especially on oxidative stress, it is essential to systematically review the existing evidence of previously published literature about Mg and oxidative stress.

## 2. Bioavailability of Mg Forms

Among the key factors influencing the effectiveness of magnesium supplementation is the chemical form in which it is administered. Magnesium supplements vary widely in their bioavailability—the proportion of Mg that is absorbed and utilized by the body—depending on their solubility and molecular composition [1].

Moreover, Mg supplements are broadly categorized into organic and inorganic compounds [9]. Organic forms, such as Mg citrate, glycinate, lactate, or malate, involve Mg chelated to organic molecules like amino acids or organic acids. These forms are generally more soluble and have higher bioavailability. Inorganic forms, such as Mg oxide, sulphate, and chloride, are bound to inorganic salts and tend to have lower solubility and absorption rates [9,10]. The chemical composition of Mg supplements significantly influences their bioavailability and physiological efficacy. A previous systematic review concluded that organic formulations of Mg are more bioavailable than inorganic ones, and the percentage of absorption is dose-dependent [10]. This systematic review also determined that not all forms are equally effective in maintaining physiological Mg levels, especially in populations with increased needs or existing deficiencies; while all Mg dietary supplements can maintain physiological levels in healthy individuals without prior deficits, this may not be assured in older adults or those with illnesses or sub-physiological Mg levels [10]. Accordingly, the Mg form used in supplementation must be personalized and adjusted to specific health considerations.

For example, Mg glycinate is often recommended for individuals with sensitive digestive systems, as it tends to cause fewer gastrointestinal side effects [11]. Mg L-threonate has gained attention for its potential cognitive benefits, attributed to its ability to cross the blood–brain barrier, though more research is needed to fully understand its efficacy in this context [12]. The following describes the main salts in which Mg is marketed and for what they are mainly used:

Mg citrate: This is one of the most bioavailable forms, with high solubility in water, leading to an efficient absorption. It is commonly used for correcting deficiencies and supporting digestion due to its mild laxative effect [13,14].

Mg glycinate: Chelated with the amino acid glycine, this form is well-absorbed and known for its calming effects, beneficial for individuals experiencing stress, anxiety, or sleep disturbances [15,16]. It is also less likely to cause gastrointestinal discomfort compared to other forms [17].

Mg taurate: It is well-absorbed [15] and usually combined with taurine, an amino acid known for its cardiovascular benefits. It stands out for its potential to support heart health by helping to regulate blood pressure, maintain a steady heart rhythm, and promote overall cardiovascular function [18,19].

Mg malate: This Mg form is chelated with malic acid, a compound involved in the Krebs cycle, the process through which the body produces energy [20]. Accordingly, this combination has been suggested as beneficial for energy production and muscle function. However, scientific evidence is limited, and further studies are needed to confirm its effects [15].

Mg L-threonate: It is notable for its ability to cross the blood–brain barrier, and is a promising option for cognitive support and neurological health [21,22,23]. Though relatively new, research suggests it may enhance memory and cognitive function. It has also been shown to have beneficial effects on sleep quality in humans [24].

Mg chloride: It has moderate bioavailability and is available in oral and topical forms. It is commonly used for muscle relaxation and electrolyte balance. The topical application allows for Mg absorption through the skin, though its effectiveness remains debated [25].

Mg sulphate: Mg sulphate [known as Epsom salt] is used to treat hypomagnesemia, prevent seizures in eclampsia, and manage constipation. It works by regulating Mg levels, with effects varying by administration route. It can cause side effects like flushing or hypotension and is contraindicated in cases of heart block or hypersensitivity [26].

Mg oxide: Contains a high percentage of elemental Mg but has low solubility, resulting in poor absorption. It is primarily used for short-term relief of constipation and acid indigestion rather than for addressing Mg deficiencies [10,27,28].

Mg lactate: Mg lactate is a well-absorbed form of Mg. Studies suggest it may aid blood pressure regulation in patients with implantable cardioverter defibrillators [29]. Research also supports its effectiveness compared to other commercial Mg supplements, making it a reliable option for maintaining adequate Mg levels [27,30]. It is also a suitable option for those looking for a form of Mg that is well tolerated by the digestive system [1].

Mg aspartate: Mg aspartate is a Mg salt of aspartic acid, commonly used as a dietary supplement to prevent and treat Mg deficiencies [1,27]. The aspartate form displays high oral bioavailability and water solubility, meaning it is efficiently absorbed by the body [31].

Mg carbonate: Mg carbonate is a common over-the-counter remedy for heartburn and indigestion caused by excess stomach acid. However, it should be used only for short-term relief, as prolonged use may lead to digestive discomfort [32].

While several forms of Mg supplements offer unique benefits, after looking at all of the types of Mg supplements that are available, evidence suggests that Mg citrate and Mg glycinate are generally considered the most effective and widely used due to their high bioavailability and minimal gastrointestinal side effects. Mg citrate is particularly favoured for its dual benefits of replenishing Mg and alleviating constipation, while Mg glycinate is preferred for its gentle nature on the digestive system, making it suitable for long-term use. Mg oxide is still widely used, though its lower bioavailability limits its effectiveness compared to other forms [11,13,14,15,16]. Each form of Mg has specific therapeutic applications, and the choice of supplement should be tailored to individual needs and health goals.

While the bioavailability of various Mg forms and their health effects has been well studied [10,16], their antioxidant capacity is attracting more interest. It acts as a cofactor for key antioxidant enzymes, such as superoxide dismutase (SOD) and glutathione peroxidase (GPx), and plays a role in maintaining cellular redox balance [33].

Recent studies have explored its effects on inflammatory pathways and mitochondrial function, both of which are closely linked to oxidative damage and chronic disease development [34,35]. While preliminary evidence suggests that Mg may contribute to antioxidant defence mechanisms, further research is needed to establish its precise role and therapeutic potential. It is essential to assess this antioxidant capacity through both habitual Mg intake in the diet and supplementation, to determine whether it has a meaningful impact.

This systematic review aims to critically assess the evidence of previously published literature related to the antioxidant effects of Mg, both from dietary intake and supplementation, considering its role in mitigating oxidative stress and promoting cellular health. In addition to reviewing studies on the bioavailability and absorption of Mg from both dietary sources and supplements, this work will include a meta-analysis to provide research work on the present issue, and a more comprehensive assessment of its potential benefits in the context of oxidative stress, which has gained increasing attention due to the growing use of Mg supplementation.

## 3. Methods

The Preferred Reporting Items for Systematic Reviews and Meta-Analyses (PRISMA) guidelines [36] were followed, and the protocol of this systematic review was registered in the Prospective International Register of Systematic Reviews (PROSPERO ID: CRD420251036570). A systematic literature search was conducted in the Medlars Online International Literature (MEDLINE) database via PubMed. The following terms from the Medical Subject Headings [MeSH] were used: “magnesium”, “eating”, “dietary supplements”, “antioxidants”, and “oxidation-reduction”. Each of the above terms was combined with Boolean AND and OR. The following Boolean search strategy was used for Mg and its antioxidant capacity or effect: <<Magnesium [MeSH Terms] AND [“eating” [MeSH Terms] OR “Oral intake” OR “Dietary Supplements” [MeSH Terms] OR [“Oral Supplements”] AND [Antioxidants [MeSH Terms]] OR [Oxidation-Reduction [MeSH Terms]]>>. This systematic review was also conducted following the PICOS (population, intervention, comparison, outcome, and study type) guidelines, a widely used approach to identify key elements of clinical evidence in systematic reviews within evidence-based medicine [37,38]. Table 1 shows the PICOS criteria used.

**Figure 1:**
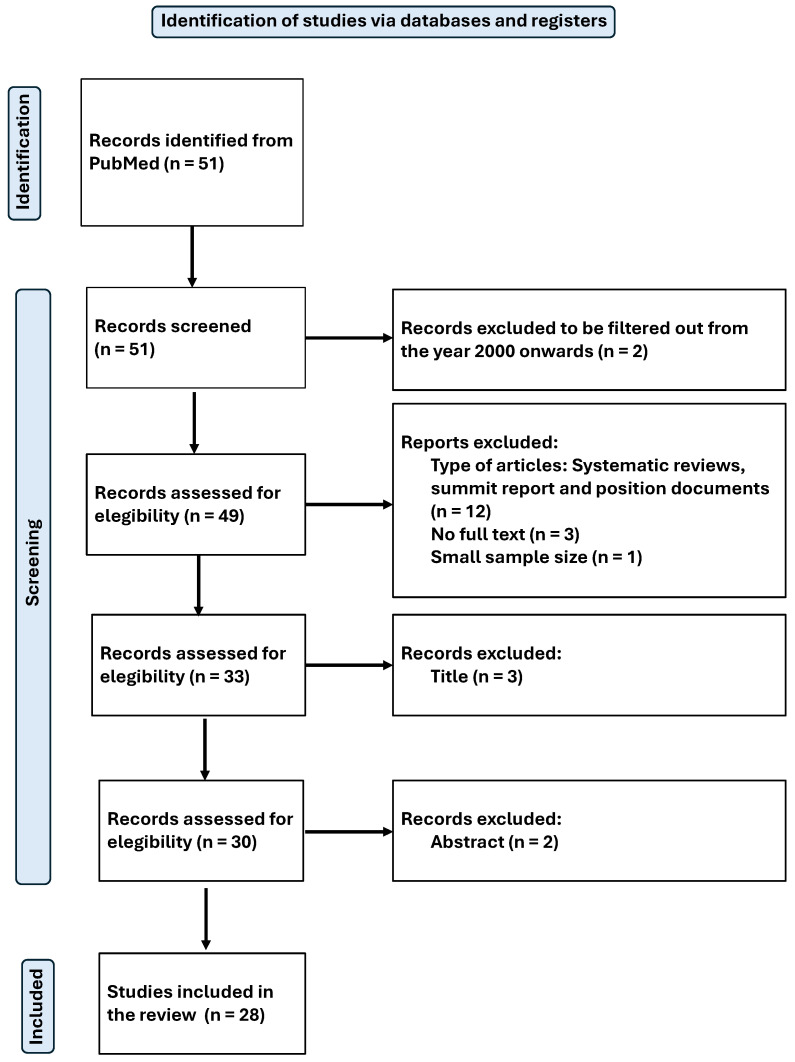
Flow-chart diagram of the systematic review.

**Figure 2:**
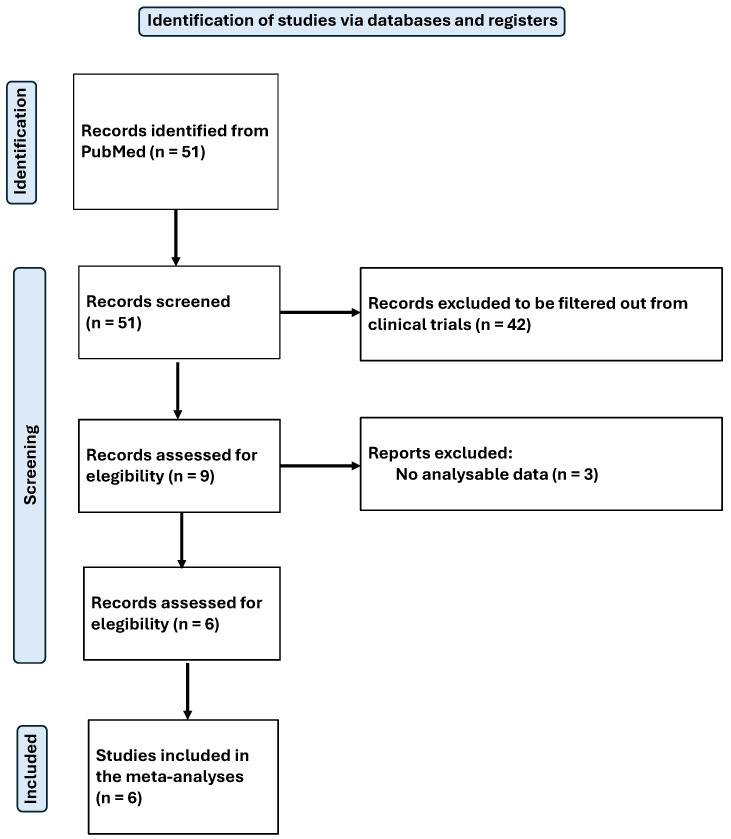
Flow-chart diagram of the meta-analysis.

The search was standardised and conducted by two independent reviewers in February 2025. The process used to identify and select articles is shown in Figure 1 for the systematic review and Figure 2 for the meta-analysis.

### 3.1. Inclusion Criteria

Original peer-reviewed research papers written in English or Spanish and published from the year 2000 to the present were considered. In total, 51 articles were identified using the search criteria detailed above.

### 3.2. Exclusion Criteria

Systematic reviews, summit reports, and position papers were excluded, as well as articles that were not complete/available, and those with a very small sample size.

### 3.3. Study Selection, Data Collection, and Extraction

The titles and then the abstracts of the articles were reviewed to determine their thematic relevance to the focus of the study. Those studies that passed this stage were subjected to a detailed full-text analysis to assess their eligibility. In cases where the information in the title and abstract were not sufficient to decide, additional context was consulted, or the full text was examined.

The selection process was conducted independently by at least two reviewers, following the criteria established by the Joanna Briggs Institute (JBI) for assessing the methodological quality of scientific articles [39] (see Supplementary Tables S1–S5). Any discrepancies between reviewers were resolved by discussion and consensus, and if disagreement persisted, a third author was involved to make the final decision.

Finally, a total of 28 articles were included in the systematic review [40,41,42,43,44,45,46,47,48,49,50,51,52,53,54,55,56,57,58,59,60,61,62,63,64,65,66,67], and 6 of them were selected for meta-analysis. All articles were related to magnesium (Mg), from which relevant data for the purpose of this review were extracted. The available results from the included studies were then meta-analysed. The main objective was to assess the physiological effects and antioxidant capacity of Mg.

The quality of analysed studies, including the risk of bias, was assessed through the Cochrane Risk of Bias-2 tool [68], as well as the Newcastle-Ottawa Scale [69] for non-randomized studies, including case-control and cohort studies, although some criticism of this has been reported [70].

### 3.4. Meta-Analysis of Data

To analyse the effect size of Mg on blood biomarkers related to oxidative stress, such as total nitrite, nitric oxide (NO), total antioxidant capacity (TAC), C-reactive protein (CRP), malondialdehyde (MDA), and reduced glutathione (GSH), including data on means and standard deviations (SD), were first collected for the control and intervention groups in each study [including both baseline and post-intervention data]. Subsequently, the mean differences and standard deviation differences were calculated to obtain the corresponding standard error (SE). The formula used was differences/square root of n. In the case of Bede et al. [46], GSH was expressed relative to haemoglobin (Hb), determined by the cyanmethemoglobin method. In all other studies, GSH was expressed as μmol/L. To convert the units, the following equation was applied: GSH (μmol/L) = GSH (μmol/gHb) × Hb (g/L), using a Hb concentration of 150 g/L.

Due to the limited number of meta-analysed studies for each parameter, the possibility of combining all parameters related to oxidative stress was explored. To achieve this and given that the various markers were expressed on different scales, standardization was performed using Z-score transformation.

The data were meta-analysed using R Studio version 2024.09.1 Build 394 “Cranberry Hibiscus”. A meta-analysis of standardized mean difference (SMD) was performed under a random-effects model. To estimate the variance between studies (τ^2^\tau^2^τ^2^), the restricted maximum likelihood (REML) method was used. Heterogeneity among studies was evaluated using Cochrane’s Q test and the I^2^ index. The Hartung–Knapp (HK) method was used to calculate confidence intervals in random-effects models to improve the precision of these estimates. The statistical significance level was defined as *p* < 0.05.

## 4. Results

### 4.1. Results of Systematic Review

The present systematic review included 28 studies which are related to the antioxidant properties of Mg [40,41,42,43,44,45,46,47,48,49,50,51,52,53,54,55,56,57,58,59,60,61,62,63,64,65,66,67]. The current results reveal several common themes reflecting the impact of Mg on health. Only four studies [48,58,65,66] used Mg supplementation alone, while the others combined it with another micronutrient. Studies were performed both in animal (13 studies) and human models (15 studies) (see Table 2).

### 4.2. Impact of Mg on Inflammation and Oxidative Stress

Being the first objective of this systematic review, several studies demonstrated that Mg supplementation, alone or in combination with zinc (Zn), vitamin C (Vit. C), vitamin E (Vit. E), or selenium, reduces inflammatory markers such as high-sensitivity C-reactive protein (hs-CRP) and interleukin-1 (IL-1) [40,41,54,55,65]. In animal studies, Mg deficiency consistently led to increased oxidative stress and inflammatory markers, evidenced by elevated lipid peroxidation, higher levels of pro-inflammatory cytokines, and mitochondrial dysfunction [42,44,45,49,50,51,53,54,56,57,58,60,66]. Conversely, Mg supplementation was associated with enhanced antioxidant enzyme activity (SOD and CAT). Altura et al. [44] found that Mg-deficient diets led to elevated lipid peroxidation and DNA fragmentation, whereas Mg repletion reversed these effects. Similarly, El-Tantawy et al. [50] demonstrated that low Mg intake resulted in higher levels of oxidative markers, which significantly decreased when Mg was supplemented.

In human studies [40,41,43,46,47,48,52,54,55,59,61,62,63,65,67], Mg supplementation, often administered with other micronutrients, was linked to improvements in oxidative balance. In the Afshar Ebrahimi et al. study [40], women with polycystic ovary syndrome who supplemented with Mg and Zn experienced a significant reduction in hs-CRP levels, IL-1, and tumour necrosis factor-α (TNF-α). With the inclusion of Mg in the supplementation, improvements were observed in the activity of antioxidant enzymes such as SOD and catalase (CAT), along with a decrease in lipid peroxidation (MDA) [43,44,45,49,53,58,60]. Alarcón-Moreno et al. [43] found that individuals undergoing non-surgical periodontal treatment and who supplemented with Mg and Zn showed a substantial increase in SOD and CAT activity compared to the control group. Additionally, Hamedifard et al. [54] demonstrated that Mg supplementation in diabetic patients not only reduced inflammatory cytokines but also improved overall oxidative stress balance by increasing glutathione levels. It has been also demonstrated that magnesium levels are related to the activation of N-methyl-d-aspartate receptors (NMDAR) of the postsynaptic hippocampal glutamatergic synapses, allowing the entrance of Na^+^ and Ca^2+^, and then modulatory effects among the regulatory processes in the body [71]. Also in endothelial cells, low Mg levels promote the acquisition of a pro-inflammatory and pro-atherogenic phenotype [72].

### 4.3. Effect on Glucose Metabolism and Lipid Profile

Several studies indicated that Mg supplementation improves glycaemic control by reducing glucose and insulin levels and enhancing insulin sensitivity [41,48,52,54,65]. Hamedifard et al. [54] found that Mg and Zn supplementation significantly lowered fasting glucose and insulin levels while increasing high-density lipoprotein (HDL) cholesterol in subjects with coronary heart disease and type 2 diabetes mellitus (T2DM). A positive impact on the lipid profile was also observed, with reductions in total and low-density lipoprotein (LDL) cholesterol levels, as well as improvements in insulin resistance index and liver function in patients with diabetes or experimental models of metabolic dysfunction [48,49,54,65]. Dou et al. [49] showed how rats with diabetes supplemented with Mg and vitamin E exhibited improved lipid profiles and reduced blood viscosity.

### 4.4. Cardiovascular Health and Vascular Function

Mg supplementation, in combination with other micronutrients, was associated with improvements in blood pressure, reducing systolic and diastolic pressure in subjects with diabetes and cardiovascular disease [52,54,55]. Farvid et al. [52] showed that a combination of Mg, Zn, vitamin C, and vitamin E resulted in a mean reduction of 7 mmHg in blood pressure among diabetic patients. Additionally, Talari et al. [65] observed a reduction in carotid intima-media thickness in haemodialysis patients who supplemented with Mg oxide for 24 weeks, suggesting a protective role in vascular health.

### 4.5. Liver Function and Protection Against Oxidative Damage

Studies in animal models showed that Mg administration reduced liver fibrosis and oxidative stress induced by toxic agents [52,60,64], while also mitigating oxidative stress related to antiretroviral therapy in HIV-transgenic rats [51]. Specifically, El-Tantawy et al. [50] found that Mg supplementation in rats with chemically induced liver fibrosis led to decreased hepatic collagen deposition and a lower expression of pro-fibrotic genes. A reduction in the expression of genes related to fibrosis and lower hepatic collagen accumulation was demonstrated.

### 4.6. Bone Health and Mineral Metabolism

A study evaluated the influence of Mg on bone metabolism, observing that its supplementation, along with calcium (Ca) and vitamin D (Vit. D), had beneficial effects on bone health and reduced metabolic complications in populations with Mg deficiency [55]. In the study, women with gestational diabetes were supplemented with Mg, Ca, Zn, and Vit. D, and experienced a reduction in inflammatory biomarkers, neonatal weight, and macrosomia rates.

### 4.7. Impact on Auditory Health and Neuromuscular Function

An association was found between adequate Mg intake and better auditory function in epidemiological and experimental studies [47,56,57]. Choi et al. [47] analysed data from NHANES 2001–2004 and found that individuals with higher dietary Mg intake had significantly lower auditory thresholds across speech and high frequencies. Additionally, in animal models of oxidative damage and aging, supplementation with Mg reduced hearing loss and improved neuromuscular function, suggesting a neuroprotective role of Mg in these conditions [56,57]. Le Prell et al. demonstrated that guinea pigs exposed to noise-induced hearing loss showed improved auditory function when supplemented with β-carotene, Vit. C and E, and Mg.

### 4.8. Effect on Anxiety and Mood

A study reflected how Mg and Zn supplementation improved symptoms of anxiety and depression, reducing scores on psychological assessment scales [54]. In this study, subjects receiving Mg and Zn showed significant reductions in Beck Anxiety Inventory and Beck Depression Inventory scores compared to the placebo group.

### 4.9. Results of Meta-Analysis

**Figure 3:**
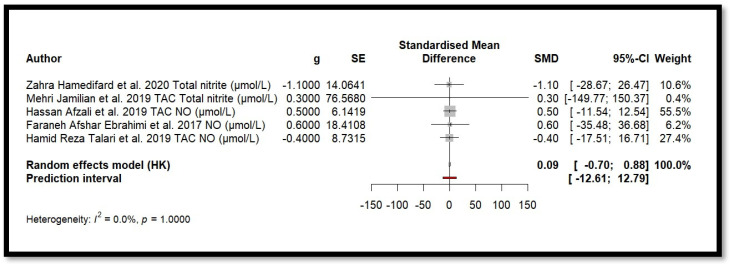
Forest plot of Mg’s effect on total nitrite and NO.

**Figure 4:**
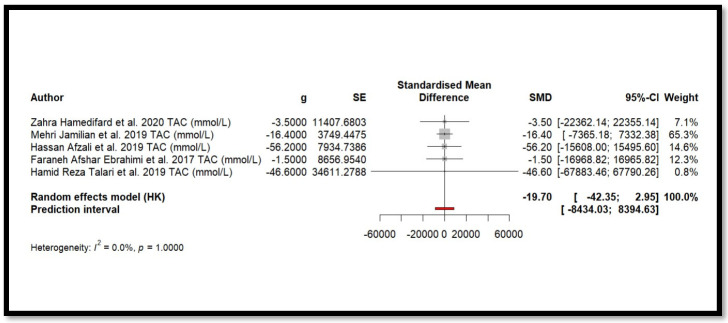
Forest plot of Mg’s effect on TAC.

**Figure 5:**
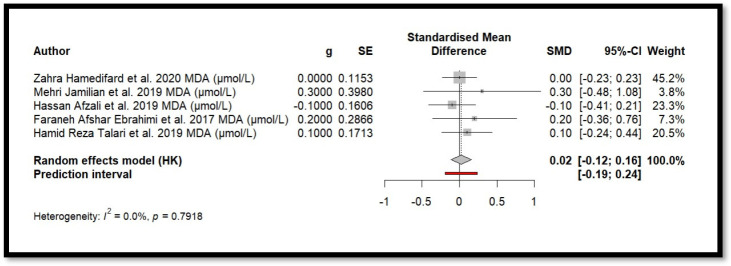
Forest plot of Mg’s effect on MDA.

**Figure 6:**
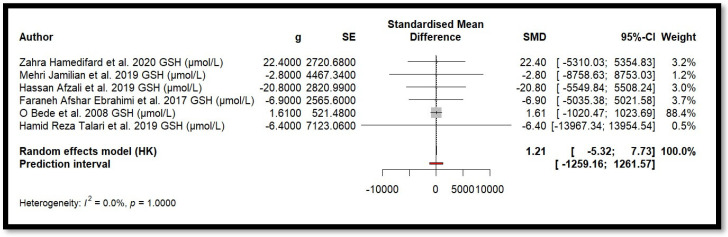
Forest plot of Mg’s effect on GSH.

Five independent meta-analyses were conducted to evaluate the effect of Mg supplementation on oxidative stress biomarkers: total nitrite and NO (Figure 3), TAC (Figure 4), MDA (Figure 5), and GSH (Figure 6). Each meta-analysis included a total of five randomized controlled trials (RCTs), except for GSH, which included six. In all forest plots, the I^2^ statistic was 0%, and the *p*-value was >0.05.

For MDA, the combined effect was 0.02 (−0.12; 0.16), with I^2^ = 0.0% and *p* = 0.7918. In the analysis of nitrogen oxides and total nitrite, the combined effect was 0.09 (−0.70; 0.88), with no observed heterogeneity (I^2^ = 0.0%, *p* = 1.000). Regarding TAC, the meta-analysis showed an SMD of −19.70 (−42.35; 2.95) with a wide confidence interval. This, combined with the low heterogeneity (I^2^ = 0.0%, *p* = 1.000), suggests that the effect of Mg on TAC remains inconclusive. Finally, for glutathione, the combined effect was 1.21 (−5.32; 7.73), with no statistical significance (*p* = 0.655) and low heterogeneity (I^2^ = 0.0%, *p* = 1.000).

In summary, all meta-analyses showed low or no heterogeneity, indicating that the reported effects across studies were homogeneous. Furthermore, the *p*-value > 0.05 suggests that the intervention did not have a significant effect on the studied parameters. Accordingly, no definitive conclusion can be drawn regarding a true relationship between the treatment and the measured outcomes, as the observed differences could be due to chance.

Additionally, for each analysed variable, the horizontal lines in the forest plots cross the null value, indicating that the obtained results are not statistically significant. Therefore, according to this meta-analysis, the effect of Mg supplementation on various oxidative stress markers remains unclear.

**Figure 7:**
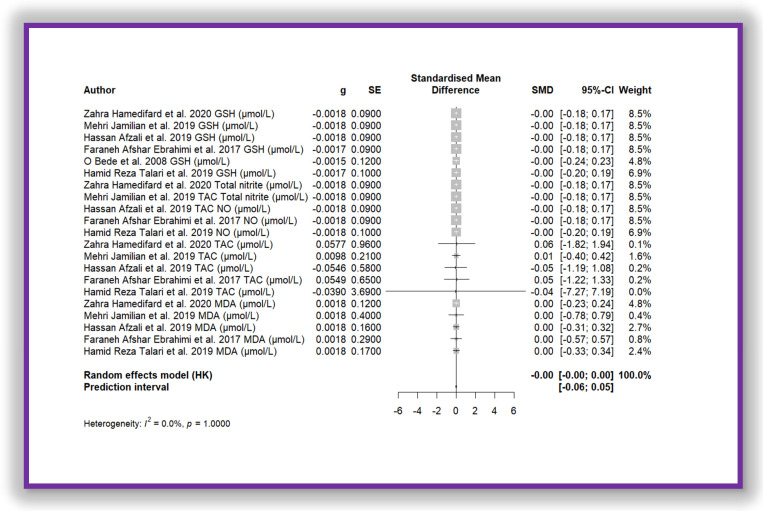
Forest plot of Mg’s effect on total biomarkers of oxidative stress.

The meta-analysis shown in Figure 7 demonstrates the same effect. This meta-analysis corresponds to the aggregation of all oxidative stress biomarker data, which were standardized using Z-score transformation. The standardized mean difference is close to zero (SMD = −0.0012 (−0.0031; 0.0007)), with a very narrow confidence interval, indicating that the observed effect is not sufficiently meaningful. Furthermore, heterogeneity is absent (I^2^ = 0.0%, *p* = 1.000), suggesting that the included studies yielded highly consistent individual results. As a result, the diamond representing the overall estimate in the forest plot is nearly imperceptible. This finding implies that there is no significant difference between the compared groups regarding the analysed oxidative stress biomarkers.

**Figure 8:**
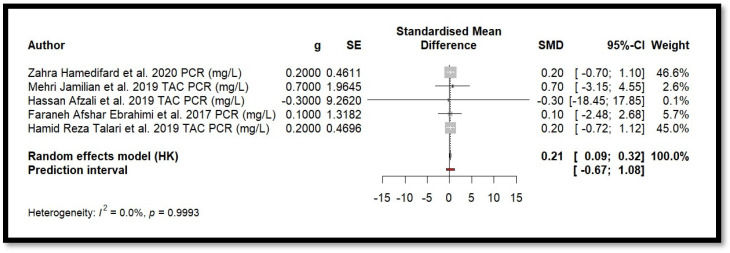
Forest plot of Mg’s effect on the inflammation biomarker (CRP).

Five randomised clinical trials were included in the meta-analysis to study the effect of Mg on CRP levels (Figure 8). Using a random-effects model adjusted by the Hartung–Knapp method, the combined effect showed SMD = 0.2066 (0.0884; 0.3247). The *p*-value associated with the combined effect was 0.008. This value indicates statistical significance. Moreover, heterogeneity among the studies was minimal (I^2^ = 0.0%, Q = 0.07, *p* = 0.999], suggesting high consistency in the results. These findings indicate a small but statistically significant positive effect of Mg in reducing CRP levels.

## 5. Discussion

The relationship between oxidative stress and inflammation is complex and bidirectional. Oxidative stress can activate several signalling pathways, such as NF-κB and MAPK, that lead to the production of pro-inflammatory cytokines, while inflammation itself can further enhance the generation of reactive oxygen species (ROS), creating a vicious cycle [35].

Magnesium seems to modulate both processes: it acts as a cofactor for antioxidant enzymes (e.g., glutathione peroxidase and superoxide dismutase) [33,73,74], and also exhibits anti-inflammatory effects by inhibiting the production of cytokines and reducing endothelial dysfunction [73,74]. Some compounds, such as zinc or selenium, share similar antioxidant and anti-inflammatory properties, suggesting that magnesium may act through the same molecular pathways [75,76]. However, the heterogeneity of biomarkers evaluated through studies limits our ability to fully characterize these mechanisms.

The findings of the current study agree with previous studies that evaluated the relationship between magnesium and biomarkers of inflammation. However, there is still some controversy. The anti-inflammatory effect described in several studies included in the systematic review [40,41,54,55,65] was also supported by meta-analyses [77,78,79], which suggested that magnesium supplementation could induce a slight but significant reduction in blood CRP levels. However, there are also other studies in which this effect is not entirely clear [80,81].

The current findings do not allow for the conclusion of a similar effect on the oxidative stress biomarkers analysed. In previous studies with animal models, it was reported that magnesium deficit contributes to oxidative stress, so that its restitution would lead to a state of physiological equilibrium through homeostatic mechanisms. Additional magnesium intake beyond basal physiological levels has not been documented to produce an improvement in the state of oxidative stress or inflammation. In contrast, in human clinical trials [40,41,43,45,46,47,48,52,54,55,59,61,62,63,65,67], Mg supplementation modulated the levels of oxidative stress biomarkers in different pathophysiological situations.

In other meta-analyses [77,79] no significant positive effect on TAC, GSH, or MDA levels was observed either. Although in the current work and in those of Heidari et al. [78] and Veronese et al. [79], a significant reduction in CRP levels was reported, the same did not occur in that of Li et al. [81]. Another point in discordance was the effect on NO levels. Both Heidari et al. [78] and Veronese et al. [79] obtained a significant increase in NO levels, while current results did not show a consistent effect of the impact of Mg supplementation on NO levels.

NO plays a role in maintaining healthy vascular function, acting as an antioxidant, but excessive NO production or disruption in its balance can lead to increased oxidative stress. However, magnesium enhances the NO role as a retrograde neurotransmitter in the NMDAR activation modulating the regulatory processes in the body [71]. Moreover, endothelial dysfunction, a crucial event in the early pathogenesis of cardiovascular diseases is linked to magnesium deficiency. In endothelial cells, low Mg levels enhance a pro-inflammatory and pro-atherogenic phenotype, contributing to the generation of a pro-oxidant state. Adequate Mg levels play a significant role in preserving cardiovascular health and preventing cardiovascular diseases [72].

The results of this systematic review support the idea that Mg supplementation contributes positively to several metabolic and cardiovascular conditions. It improved glycaemic control and insulin sensitivity, reducing insulin resistance and fasting glucose levels. It also resulted in improvements in the lipid profile in terms of reduced total cholesterol and LDL, and an increase in HDL. However, in the context of cardiovascular health, Mg supplementation has been shown to be effective in lowering blood pressure in patients with diabetes and cardiovascular disease. Moreover, it has been related to a decrease in carotid intima-media thickness in haemodialysis patients. Current findings agree with those reported by El-Derawi et al. [82], who observed how Mg supplementation reduces insulin resistance and improves glycaemic control indicators in patients with T2DM. On the other hand, Mg supplementation with Zn also appears to have beneficial effects in patients with T2DM and coronary heart disease, improving lipid profile and fasting plasma glucose levels [54].

Other studies suggest that Mg could play an important role in liver diseases [50,83], both in humans and animal models [84]. It is observed that Mg could have hepatoprotective effects due to the reversal of liver damage caused by fibrosis induction. However, further human clinical studies are needed to confirm these effects and explore the underlying mechanisms.

This systematic review show that an adequate intake of Mg, both through diet and supplementation, can contribute significantly to overall well-being and the prevention of several health conditions related to bone, hearing, neuromuscular, and mental health [85,86,87,88]. Some research demonstrated the efficacy of Mg in the prevention and treatment of hearing damage, particularly noise-induced hearing loss, in both animal [85] and human [86] studies. Furthermore, studies on bone health cannot be attributed to Mg alone, as its supplementation or intake is often accompanied by other micronutrients [85]. A previous systematic review concluded that studies on Mg and its effect on psychiatric disorders were not entirely consistent, either as a supplement or alone, yet low plasma Mg levels were observed in people with depression [84].

### Strengths, Limitations, and Future Considerations

The main strength of this study is that it has systematically reviewed previously published literature related to the antioxidant effects of Mg on oxidative stress, but this work also includes a meta-analysis to provide a more comprehensive assessment of its potential benefits in the context of oxidative stress.

Despite this, the current study has several limitations shared by many of the studies analysed. The number of studies included in the meta-analysis was small and they applied small sample sizes with relatively short intervention periods, which limits the generalizability of the findings. Moreover, most of the eligible articles did not administer Mg alone, but in combination with other micronutrients, so it cannot know whether the effect is due to magnesium alone, to the other components, or to the synergy of both. Neither were the same doses of Mg administered nor was the same salt used.

It should also be considered that the effects of magnesium as a supplemental essential nutrient may be influenced by the individual overall nutritional status. Individuals with magnesium deficiencies would be more positively impacted by supplementation than those with adequate magnesium levels. Accordingly, future studies should take this factor into account in their design.

Finally, it should be noted that the participants in the different studies suffered from different diseases. Therefore, future studies should focus on clinical trials with more homogeneous designs and a larger sample size to evaluate more precisely the impact of magnesium on inflammation and oxidative stress. Moreover, it would be advisable to explore the influence of different formulations and doses of magnesium to determine which may be more effective.

## 6. Conclusions

The findings of the current review and meta-analysis suggest that magnesium supplementation could have a slight positive effect on the reduction of serum CRP, but not on some biomarkers related to oxidative stress. In any case, this impact remains uncertain. It is possible that the known antioxidant activity of magnesium has been demonstrated to a greater extent in experimental studies, that it is linked to the regulation of other types of oxidative stress biomarkers than NO, TAC, MDA, or GSH, or that it is linked to the anti-inflammatory effect of Mg itself, as chronic inflammation may increase the release of free radicals [89]. Therefore, further clinical research is needed to explore the effects of magnesium in different specific contexts and to determine whether these effects can be translated into human health benefits.

## Figures and Tables

**Table 1:** PICOS used.

Patients	Intervention	Comparison	Outcomes	Study Type
General population, rats and mice with or without pathology	Oral Mg intake or supplementation and antioxidant capacity	Physiological effect and antioxidant capacity of oral intake or supplementation with or without Mg	Physiological, biochemical, or clinical parameters	Cross-sectional, longitudinal, case-control, cohort, and controlled trials

**Table 2:** Descriptive table of the studies included in the systematic review.

Reference	Study Design	Sample Subjects and Groups	Duration of Intervention	Mg Intervention	Parameters Analysed	Results/Conclusion
Afshar Ebrahimi et al. 2017 [40]	Randomised clinical trial	N total = 60 (two groups). Women with polycystic ovary syndrome.	12 weeks	CG (n = 30): Placebo. IG (n = 30): 250 mg Mg oxide + 220 mg Zn sulphate.	Mg in blood. Gene expression.	Mg + Zn reduced inflammation (hs-CRP), increased antioxidants, and decreased interleukin-1 and tumour necrosis factor-α expression.
Afzali et al. 2019 [41]	Randomised clinical trial	N total = 57 (2 groups). Subjects with grade 3 diabetic foot ulcer.	12 weeks	CG (n = 28): placebo. IG (n = 29): 250 mg of Mg + 400 IU Vit. E.	Insulin, hs-CRP, and MDA in blood	Mg + Vit. E improved healing, glycaemic control, lipid profile, and inflammatory markers.
Ahokas et al. 2005 [42]	Quasi-experimental study	N total = 25 (5 groups) male Sprague-Dawley rats.	4 weeks	G1. CG (n = 5): Untreated rats, no surgery. G2. ALDOST (n = 5): Uninephrectomy, standard diet (20–40 mg/kg Mg), water with 1% NaCl and 0.4% KCl and aldosterone (0.75 µg/h). G3 (n = 5): ALDOST + Mg (40–60 mg/kg). G4 (n = 5): ALDOST + amlodipine (10 mg/kg/day). G5 (n = 5): ALDOST + NAC 200 mg/kg/day.	Blood Mg and vascular inflammation.	Supplementation with Mg (G3) and NAC (G5) reduced vascular inflammation and oxidative stress.
Alarcón-Moreno et al. 2024 [43]	Quasi-experimental study	N total = 39 (2 groups). Subjects with non-surgical periodontal treatment and T2DM.	30 days	CG (N = 19): Placebo. IG (N = 20): 500 mg Mg oxide + 50 mg Zn gluconate	Oxidative biomarkers (MDA and antioxidant enzymes).	Mg + Zn significantly increased SOD and CAT activity compared to non-surgical periodontal treatment alone (CG).
Altura et al. 2009 [44]	Quasi-experimental study	Total N = 89 (5 groups). Wistar rats.	21 days	CG: No Mg deficiency and no treatment. G1 MgD: Mg-deficient. G2 MgD + 15 mg Mg/L in water. G3 MgD + 40 mg Mg/L in water. G4 MgD + 100 mg Mg/L in water.	Mg in serum, cell damage (fragmented DNA), lipid peroxidation, caspase-3 activation, sphingomyelin, and phosphatidylcholine.	Mg deficiency increased lipid peroxidation and DNA fragmentation; supplemented Mg, even at low doses, was protective against cardiovascular damage and oxidative stress.
Bassey et al. 2022 [45]	Quasi-experimental study	N total = 30 rats (5 groups).	No data	G1 (N = 6): Non-diabetic. G2 (N = 6): Untreated diabetic group. G3 (N = 6): Mg 400 mg/kg + Ca 400 mg/kg × body weight. G4 (N = 6): Vit. C 100 mg/kg + Vit. E 100 mg/kg × body weight. G5 (N = 6): Mg 400 mg/kg + Ca 400 mg/kg + Vit. C 100 mg/kg + Vit. E 100 mg/kg. They were allowed free access to food and water.	TAC, NO, MDA, and seminal quality.	Mg + Ca + Vit. C + E (G5) improved semen and antioxidant parameters; Vit. C + E (G4) had the greatest effect.
Bede et al. 2008 [46]	Randomised clinical trial	N total = 40 asthmatic children (from 4 to 16 years old).	12 weeks	CG: Placebo. IG: <7 years old received 200 mg and >7 years old 290 mg Mg citrate.	-Oxidized and reduced glutathione (GSSG and GSH). Oxyhaemoglobin, methaemoglobin (metHb), and haemochromes. -Bilirubin levels. -GSH stability tests.	Mg increased GSH and reduced metHb and haemochrome in the treated group vs. placebo.
Choi et al. 2014 [47]	Prevalence and incidence study	N total = 2592 subjects (20–69 years old).	Extracted from NHANES 2001–2004	Dietary intake of Mg (non-supplemented) determined by 24 h dietary reminder.	Dietary b-carotene, Vit. C, Vit. E, and Mg. Audiometry examination.	Higher intakes of β-carotene, Vit. C, and Mg were associated with lower (better) pure tone averaged auditory thresholds at both speech and high frequencies.
Djurhuus et al. 2001 [48]	Quasi-experimental study	N total = 15 subjects (2 groups).	24 weeks	CG (N = 5): Healthy subjects. IG (N = 10): Type I diabetics received an intravenous dose of 30 mmol MgSO_4_ in 500 mL 0.9% NaCl, followed by 500 mg oral MgO, twice daily.	Serum Mg, muscle Mg, renal Mg excretion, serum lipids, glucose uptake, and Mg intake.	Mg supplementation increased muscle Mg by 5%, reduced cholesterol and LDL, and decreased glucose uptake.
Dou et al. 2009 [49]	Randomised clinical trial	N total = 98 Wistar rats with diabetes.	8 weeks	G1 (N = 15): Control, no supplementation. G2 (N = 17): 0.5 g/kg Vit. E. G3 (N = 16): 0.6 g/kg Mg. G4 (N = 16): 1.2 g/kg Mg. G5 (N = 17): 0.5 g/kg Vit. E + 0.6 g/kg Mg. G6 (N = 17): 0.5 g/kg Vit. E + 1.2 g/kg Mg.	Oxidative stress, lipid profile, and blood viscosity.	Mg + Vit. E improved the lipid profile and reduced blood viscosity; they may affect lipid peroxidation, but did not directly influence it.
El-Tantawy et al. 2017 [50]	Quasi-experimental study	N total = 36 Wistar rats (5 groups).	1 month	CG (N = 6): NOT CCl_4_-induced liver fibrosis, healthy rats. G1 (N = 6): CCl_4_ + No treatment. G2 (N = 6): CCl_4_ + Mg 20 mg/150 gm bw. G3 (N = 6): CCl_4_ + Mg 20 mg + 17 mg Sacch. cerevisiae/150 gm bw. G4 (N = 6): CCl_4_ + Mg 20 mg, Ca 165 mg, Vit. D3 82 IU, and 37 mg Vit. C/150 g bw. G5 (N = 6): CCl_4_ + Silymarin at a dose of 50 mg/kg bw.	(1) Collagen I, transforming growth factor β1, platelet-derived growth factor-C, and nuclear factor kappa-β gene expression. (2) Levels of hepatic collagen (hydroxyproline). (3) Oxidative and antioxidant stress markers: MDA, NO, and GSH. SOD and GST activities. (4) Histopathological examination.	Mg reduced liver fibrosis and oxidative stress; the G5 formulation showed the greatest inhibition of collagen I, transforming growth factor β1, and hepatic hydroxyproline.
ElZohary et al. 2019 [51]	Quasi-experimental study	N total = Nº not specified, rats (8 groups).	18 weeks	G.A1: Control, normal Mg group. G.A2: Control + cART treatment. G.A3: HIV-Tg rats alone. G.A4: HIV-Tg rats + cART treatment (normal Mg). G.B1: Control + high Mg. G.B2: Control + cART treatment + high Mg. G.B3: HIV-Tg rats + high Mg. G.B4: HIV-Tg rats + cART treatment + high Mg. Groups A1–A4 were fed a Mg-normal (0.1% MgO/kg food) diet and groups B1-B4 received a Mg-supplemented (0.6% MgO/kg food) diet.	Genes related to oxidative/nitrosative stress and lipogenesis are analysed by RT-PCR in the liver. Plasma biomarkers (8-isoprostane, nitrotyrosine, RBC-GSSG) and triglyceride and cholesterol levels are also measured.	Mg supplementation in HIV-1-transgenic rats attenuated metabolic and oxidative/nitrosative stress induced by cART. It normalized antioxidant gene expression (HmOX1 and GST), reduced iNOS levels, and improved lipid profiles.
Farvid et al. 2004 [52]	Randomised clinical trial	N total = 69 diabetic subjects (4 groups).	3 months	CG (N = 18): Placebo. G1 (N = 16): 200 mg Mg + 30 mg Zn. G2 (N = 18): 200 mg Vit. C + 150 mg Vit. E. G3 (N = 17): Mg + Zn + Vit. C + E (above indicated quantities).	Blood pressure and biochemical parameters such as serum potassium and MDA were measured.	In G3, there was a significant reduction of systolic (−8 mmHg, *p* < 0.05), diastolic (−6 mmHg, *p* < 0.05), and mean (−7 mmHg, *p* < 0.05) blood pressure; increase in serum potassium (*p* < 0.05); and decrease in MDA (*p* < 0.05).
Glombowsky et al. 2018 [53]	Quasi-experimental study	N total = 10 calves (2 groups).	30 days	CG: (N = 5): Non-supplemented. IG (N = 5): Two intramuscular doses (3 mL) of a commercial mineral complex on days 2 and 14 post-birth. Each 100 mL of the product had NaGly (14 g), NaH_2_PO_4_ (20.1 g), CuCl_2_ (0.4 g), KCl (0.6 g), MgCl_2_ (2.5 g), Na_2_SeO_3_ (0.24 g), and sterile H_2_O.	Blood and faecal samples, body weight and body temperature, blood counts, seric biochemistry, SOD, CAT, and GPx activity.	Mineral supplementation improved antioxidant activity and reduced infections in newborn calves.
Hamedifard et al. 2020 [54]	Randomised clinical trial	N total = 60 subjects with coronary heart disease and T2DM (2 groups).	12 weeks	CG (N = 30): Placebo. IG (N = 30): Oral supplementation: 250 mg of Mg oxide + 150 mg of Zn sulphate.	Blood samples (glucose, insulin, CRP, HDL, and TAC) and test for anxiety (BAI) and depression (BECK).	Mg + Zn reduced glucose, insulin, and CRP; increased HDL and TAC; and improved anxiety and depression.
Jamilian et al. 2019 [55]	Randomised clinical trial	N total = 60 women with gestational diabetes (2 groups).	6 weeks	CG (N = 30): Placebo. IG (N = 30): 100 mg Mg, 4 mg Zn, 400 mg Ca, and 200 IU Vit. D supplements.	Blood samples (oxidative stress biomarkers, hs-CRP, and nitrites) and pregnancy variables.	Supplementation reduced hs-CRP and MDA, increased TAC, and decreased neonatal weight and macrosomia rate.
Le Prell et al. 2011 [56]	Randomised clinical trial	N total = 25 guinea pigs (2 groups).	6 days	CG (N = 9): Saline and vegetable oil. IG (N = 16): β -carotene and vitamins C and E + 2.85 mmol/kg of Mg sulphate.	Plasma levels, noise response, and ear hair cells.	Mg + antioxidants reduced noise-induced hearing loss.
Le Prell et al. 2011 [57]	Quasi-experimental study	N total = 31 CBA/J mice (3 groups).	28 days	CG (N = 16): Control diet (CD). IG-A (N = 8): CD + Mg 2656 + β-carotene 77 + ascorbic acid 2250 + α-tocopherol 863. IG-B (N = 7): CD + Mg 4500 + β -carotene 224 + ascorbic acid 3600 + α -tocopherol 2650. Quantities are indicated per mg/kg of pellet chow.	Permanent hearing threshold loss, survival of hair cells, type II fibroblasts loss, and cell density.	Antioxidant supplementation did not protect hair cells, but reduced hearing loss and showed potential in oxidative stress-related diseases.
Liu et al. 2007 [58]	Quasi-experimental study	N total = 96 (2 groups) male Arbor Acre broiler chickens on reactive oxygen species (ROS).	6 weeks	CG (n = 48): Diet with 2.4 g Mg/kg dry matter. Low Mg group (n = 48): Diet with 1.2 g Mg/kg dry matter.	Oxidative stress: ROS, MDA, and GSH. Mitochondrial activity. Concentration of Mg, Fe, Ca, and fatty acids in muscle.	Low Mg increased ROS, MDA, and mitochondrial activity in muscles.
Manuel y Keenoy et al. 2000 [59]	Quasi-experimental study	N total = 93 (2 groups) patients with chronic fatigue.	3 months	G1: No Mg deficiency and no treatment group (n = 49). G2: Mg deficiency group (n = 44, 20% or more retention of intravenously infused Mg): Mg 10 mg/kg/day.	Antioxidant capacity in plasma, GSH, Vit. E, and lipid peroxidation (TBARS in non-HDL lipoproteins)	Mg supplementation in Mg-deficient patients improved Mg stores, increased Vit. E, and reduced oxidative stress.
Markiewicz-Górka et al. 2011 [60]	Randomised clinical trial	N total = 40 mice (5 groups).	3 months	G0 CG (n = 8): No alcohol intoxication. G1 ethanol group (n = 8): Intoxicated with alcohol (15% ethanol in drinking water). G2 Et + Mg group (n = 8): Intoxicated with alcohol + Mg (100 mg/L water). G3 Et + Se group (n = 8): Intoxicated with alcohol + Se (0.4 mg/L water). G4 Et + Mg + Se group (n = 8): Intoxicated by alcohol + Mg + Se.	Oxidative stress: Serum TAS, GPx activity in liver, reduced/oxidised glutathione (GSH/GSSG) ratio in liver. Lipid peroxidation. Liver histopathology.	Group Et + Mg + Se reduced liver damage and improved TAS and GPx.
McKeever et al. 2002 [61]	Cohort study	2633 adults (1991) and 1346 adults (2000).	No data	Cross-sectional (comparing data from 1991 and 2000) and longitudinal (analysing the change in lung function (FEV1) between the two years) analyses were performed.	Lung function was measured using forced expiratory volume in 1 s (FEV1). Dietary intake of Mg and Vit. C via the food frequency questionnaire.	Higher Vit. C and Mg intake → better lung function (FEV1) in cross-sectional analyses. At 9-year analysis → only Vit. C was associated with less lung function loss. Each 100 mg/day of Vit. C → reduced FEV1 decline by 50.8 mL (95% CI: 3.8–97.9). Mg had no impact on long-term FEV1 decline.
Morais et al. 2016 [62]	Prevalence and incidence study	N total = 83 women.	No data	CG (n = 52): Women with BMI = 18.5 and 24.9 kg/m^2^. Obese group (n = 31): Women with BMI = 30 and 39.9 kg/m^2^. Dietary Mg calculated by 3-day log.	Dietary intake of Mg. Blood Mg concentration (plasma and erythrocyte). Oxidative stress and lipid peroxidation: TBARS.	Obese women had higher oxidative stress and lipid peroxidation (*p* < 0.05). Positive correlation between erythrocyte Mg levels and TBARS in the obese group (*p* = 0.021).
Picado et al. 2001 [63]	Case–control study	Total N = 239 subjects.	No data	GC (n = 121): Healthy subjects. Group with asthma (n = 118): Patients with asthma diagnosis. Comparison between groups: - Asthmatics vs. healthy subjects. - Different levels of asthma severity.	Blood levels (plasma and serum) and dietary intake (food frequency questionnaire) of Vit. C, E, A, Mg, Zn, and Se. Antioxidant activity: GSH-Px. Asthma severity according to four severity groups.	There were no differences in the levels of vitamins (C, E, and A), Se, Mg, and Zn between the two groups. Asthma severity was not related to diet or levels of these micronutrients. Patients with severe asthma had lower GSH-Px activity. This suggests that more severe asthmatics may have lower antioxidant capacity.
Singh et al. 2007 [64]	Quasi-experimental study	N total = 48 Sprague-Dawley adult rats (8 groups).	No data	Group I (n = 6): Served as normal control (vehicle only). Group II (n = 6): Treated as experimental control (dimethyl mercury (DMM) 10 mg/kg, po, once only). Group III (n = 6): DMM + NAC + Zn. Group IV (n = 6): DMM + NAC + Se. Group V (n = 6): DMM + NAC + Mg. Group VI (n = 6): DMM + DPA + Zn. Group VII (n = 6): DMM + DPA + Se. Group VIII (n = 6): DMM + DPA + Mg.	Biomarkers of liver and kidney damage: AST, ALT, ALP, LDH, GGT, bilirubin, and creatinine. Oxidative stress: Lipid peroxidation and GSH. Acetylcholinesterase activity. Efficacy of treatments with chelators and supplements.	NAC + Se and DPA + Mg treatments were the most effective in reducing oxidative damage and restoring biochemical parameters.
Talari et al. 2019 [65]	Randomised clinical trial	N total = 54 patients with diabetes on haemodialysis (HD).	24 weeks	CG (n = 27): Placebo. IG (n = 27): Supplementation with Mg oxide 250 mg/d.	Carotid intima-media thickness. Glycaemic control: Serum insulin levels, insulin resistance index, percentage of glycated haemoglobin, and insulin sensitivity index. Cardiometabolic risk markers: Total cholesterol, LDL, hs-CRP, MDA, and TAC.	Mg supplementation for 24 weeks in patients with diabetes on haemodialysis improved arterial health, better controlled glucose, reduced inflammation and oxidative stress, and improved lipid profile.
Tatarkova et al. 2020 [66]	Quasi-experimental study	N total = 20 mice (4 groups).	2 weeks	G1 (n = 5): Normal Mg diet and SLC41A1 +/+ genotype (normal control). G2 (n = 5): Low Mg diet and SLC41A1 +/+ genotype (low Mg diet control). G3 (n = 5): Normal Mg diet and SLC41A1-/- genotype (knockout genotype with normal diet). G4 (n = 5): Low Mg diet and SLC41A1-/- genotype (knockout genotype with low Mg diet).	Krebs cycle enzymes: Aconitate hydratase, isocitrate dehydrogenase, and α-ketoglutarate dehydrogenase. Electronic transport chain complexes: CI, CII, CIII, CIV, and CV.	Dietary Mg content and the functionality of the SLC41A1 exchanger influence energy production in cardiac mitochondria, affecting the activity of key enzymes in the Krebs cycle and the electron transport chain.
Wolters and Hahn 2003 [67]	Randomised clinical trial	N total = 220 older women (two groups).	6 months	CG (n = 109): Placebo. IG (n = 111): Supplementation of coenzyme Q10 + multivitamins + Se + Mg (50 mg)	Serum concentrations of coenzyme Q10.	Supplementation increased serum coenzyme Q10 levels by 106% in the intervention group and 31% in the control group.

ALDOST: aldosterone; ALP: alkaline phosphatase; ALT: alanine aminotransferase; AST: aspartate aminotransferase; cART: Atazanavir-Ritonavir + Truvada—HIV drug combination; CuCl_2_: copper chloride; DMM: dimethyl mercury; DPA: D-penicillamine; FEV1: forced expiratory volume in 1 s; GPx: glutathione peroxidase; GST: glutathione S-transferase; GSSG: oxidized glutathione; HmOX1: heme oxygenase-1; IG: intervention group; iNOS: inducible nitric oxide synthase; KCl: potassium chloride; LDH: lactate dehydrogenase; MgCl_2_: magnesium chloride; metHb: methaemoglobin; NaCl: sodium chloride; NaGly: sodium glycerophosphate; NaH_2_PO_4_: monosodium phosphate; Na_2_SeO_3_: sodium selenite; NAC: N-acetylcysteine; ROS: reactive oxygen species; Se: selenium; TAS: total antioxidant status; TBARS: thiobarbituric acid reactive substances.

## Data Availability

There are restrictions on the availability of the data used for this trial due to the signed consent agreements around data sharing, which only allow access to external researchers for studies adhering to the project’s purposes. Requestors wishing to access the trial data used in this study can make a request to pep.tur@uib.es.

## Supplementary Materials

The following supporting information can be downloaded at https://www.mdpi.com/article/10.3390/antiox14060740/s1. Table S1: Evaluation of the methodological quality of studies according to JBI Critical appraisal checklist for randomized controlled trials; Table S2: Evaluation of the methodological quality of studies according to JBI Critical appraisal checklist for case control studies; Table S3: Evaluation of the methodological quality of studies according to JBI Critical appraisal checklist for prevalence studies; Table S4: Evaluation of the methodological quality of studies according to JBI Critical appraisal checklist for cohort studies; Table S5: Evaluation of the methodological quality of studies according to JBI Critical appraisal checklist for non-randomized experimental studies.

## Abbreviations

Ca (calcium), CAT (catalase), CRP (C-reactive protein), GSH (reduced glutathione), Hb (hemoglobin), MDA (malondialdehyde), MeSH (medical subject headings), Mg (magnesium), NO (nitric oxide), SD (standard deviation), SE (standard error), SMD (standard mean difference), SOD (superoxide dismutase), T2DM (type 2 diabetes mellitus), TAC (total antioxidant capacity), Vit. D (vitamin D).
