# Supplementary material for: Unlocking the Power of Magnesium: A Systematic Review and Meta-Analysis Regarding Its Role in Oxidative Stress and Inflammation
Source: Antioxidants (Basel). 2025 Jun 16;14(6):740. doi: 10.3390/antiox14060740 (PMC12189353; doi:10.3390/antiox14060740)
Supplement: Supplementary file 1 [file antioxidants-14-00740-s001.zip › antioxidants-3645395-supplementary.pdf]

Supplementary Table S1. Evaluation of the methodological quality of studies according to JBI Critical appraisal checklist for randomized controlled trials.

**Reference**	**Q1**	**Q2**	**Q3**	**Q4**	**Q5**	**Q6**	**Q7**	**Q8**	**Q9**	**Q10**	**Q11**	**Q12**	**Q13**	**TOTAL YES**
HamedifarZ, et al., 2020	YES	YES	YES	YES	YES	YES	NO	YES	YES	YES	U	YES	YES	11
Jamilian M,et al., 2019	YES	YES	YES	YES	YES	YES	U	YES	YES	YES	YES	YES	YES	12
Talari HR,et al., 2019	YES	YES	YES	YES	YES	YES	YES	YES	YES	YES	YES	YES	YES	13
Afzali H,et al., 2019	YES	YES	YES	YES	YES	YES	YES	YES	YES	YES	YES	YES	YES	13
Afshar Ebrahimi F,et al., 2018	YES	YES	YES	YES	YES	YES	U	YES	YES	YES	YES	YES	YES	12
Le Prell CG,et al., 2011	YES	U	YES	U	U	YES	YES	YES	YES	YES	YES	YES	YES	10
Markiewicz-Gorka l, et al., 2011	YES	YES	YES	N/A	U	YES	U	YES	YES	YES	YES	YES	YES	10
Dou M, et al., 2009	YES	U	YES	YES	U	YES	U	YES	YES	YES	YES	YES	YES	10
Bede O, et al., 2008	YES	YES	YES	YES	YES	YES	YES	YES	YES	U	YES	YES	YES	12
Farvid MS,et al., 2004	YES	U	YES	YES	YES	YES	U	YES	YES	YES	YES	YES	YES	11
Wolters M and Hahn A., 2003	YES	YES	YES	YES	YES	YES	YES	YES	YES	YES	YES	YES	YES	13

Supplementary Table S2. Evaluation of the methodological quality of studies according to JBI Critical appraisal checklist for case control studies.

Reference	Q1	Q2	Q3	Q4	Q5	Q6	Q7	Q8	Q9	Q10	TOTAL YES
Picado C, et al., 2001	YES	YES	YES	YES	YES	YES	YES	YES	YES	YES	10

Supplementary Table S3. Evaluation of the methodological quality of studies according to JBI Critical appraisal checklist for prevalence studies.

Reference	Q1	Q2	Q3	Q4	Q5	Q6	Q7	Q8	Q9	TOTAL YES
Morais JBS, et al., 2017	YES	YES	YES	YES	YES	YES	YES	YES	N/A	8
Choi YH,et al., 2014	YES	YES	YES	YES	YES	YES	YES	YES	YES	9

Supplementary Table S4. Evaluation of the methodological quality of studies according to JBI Critical appraisal checklist for cohort studies.

**Reference**	**Q1**	**Q2**	**Q3**	**Q4**	**Q5**	**Q6**	**Q7**	**Q8**	**Q9**	**Q10**	**Q11**	**TOTAL YES**
**Mckeever TM,et al., 2002**	YES	YES	YES	U	U	YES	YES	YES	YES	YES	YES	9

Supplementary Table S5. Evaluation of the methodological quality of studies according to JBI Critical appraisal checklist for non-randomized experimental studies.

Reference	Q1	Q2	Q3	Q4	Q5	Q6	Q7	Q8	Q9	Total Yes
**Alarcón-Moreno JC, et al., 2024**	YES	YES	YES	YES	YES	YES	YES	YES	YES	9
**Tatarkova Z, et al., 2020**	YES	YES	YES	YES	NO	YES	YES	N/A	YES	7
**Bassey IE, et al., 2022**	YES	YES	YES	YES	NO	YES	YES	N/A	YES	7
**ElZohary L, et al., 2019**	YES	YES	YES	YES	U	YES	YES	N/A	YES	7
**Glombowsky P, et al., 2018**	YES	YES	YES	YES	YES	YES	YES	NO	YES	8
**El-Tantawy WH, et al., 2017**	YES	YES	YES	YES	U	YES	YES	N/A	YES	7
**Le Prell CG, et al., 2011**	YES	YES	YES	YES	YES	YES	YES	NO	YES	8
**Altura BM, et al., 2009**	YES	YES	YES	YES	NO	YES	YES	NO	YES	7
**Singh V, et al., 2007**	YES	YES	YES	YES	NO	YES	YES	NO	YES	7
**Ahokas RA, et al., 2005**	YES	YES	YES	YES	NO	YES	YES	N/A	YES	7
**Manuel and Keenoy B, et al., 2000**	YES	NO	YES	YES	YES	YES	YES	NO	YES	7
**Liu YX,et al., 2007**	YES	YES	YES	YES	U	YES	YES	U	YES	7
**Djurhuus MS,et al., 2001**	YES	YES	YES	YES	YES	YES	YES	YES	YES	9
